# Curve Progression After the Termination of Bracing for Adolescent Idiopathic Scoliosis: Usefulness of Combining the Proximal Femur Maturity Index (PFMI) and Risser Staging

**DOI:** 10.7759/cureus.73395

**Published:** 2024-11-10

**Authors:** Hisakazu Shitozawa, Haruo Misawa, Koji Uotani, Tomoko Tetsunaga, Kensuke Shinohara, Yoshiaki Oda, Masataka Ueda, Ryo Takatori, Kazutaka Yamashita, Toshifumi Ozaki

**Affiliations:** 1 Department of Orthopaedic Surgery, Section of Medicine, Division of Medicine, Dentistry and Pharmaceutical Sciences, Graduate School of Medicine, Dentistry and Pharmaceutical Sciences, Okayama University, Okayama, JPN; 2 Department of Orthopaedic Surgery, Ryusou Orthopaedic Hospital, Okayama, JPN; 3 Department of Orthopaedic Surgery, Okayama University Hospital, Okayama, JPN; 4 Department of Sports Medicine, Faculty of Medicine, Dentistry and Pharmaceutical Sciences, Okayama University, Okayama, JPN; 5 Department of Orthopaedic Surgery, Faculty of Medicine, Dentistry and Pharmaceutical Sciences, Okayama University, Okayama, JPN

**Keywords:** adolescent idiopathic scoliosis (ais), height growth, proximal femur maturity index, risser staging, skeletal maturation

## Abstract

Background

The brace therapy for adolescent idiopathic scoliosis (AIS) typically ends upon the end of growth. However, determining the timing of growth cessation can be challenging. The purpose of this study was to evaluate the utility of the proximal femur maturity index (PFMI), which can be assessed simultaneously with Risser staging without requiring additional radiation exposure, in determining the appropriate timing to terminate bracing. To achieve this, we investigated the relationship between skeletal maturity at the end of bracing, post-bracing curve progression, and height growth in patients who had been successfully treated with a brace.

Methods

Between April 2010 and March 2021, a total of 84 female patients with AIS who started bracing at our hospital with an initial Cobb angle of 20-40 degrees were included. All patients were followed for at least one year after brace termination. Height and radiographic parameters (Risser staging, PFMI, Cobb angle) were retrospectively collected.

Results

At the end of the bracing period, patients were categorized into Risser stage 4 (85.7%) and 5 (14.3%). By the last follow-up, patients with Risser stage 4 experienced an average main curve progression of 1.8°, whereas those with Risser stage 5 had an average progression of −0.3° (*P* = 0.03). Patients with Risser stage 4 were further divided into PFMI grade 5 (59.7%) and 6 (40.3%). Significant curve progression was observed in patients with PFMI grade 5 (average: 3.0°) compared to grade 6 (average: -0.6°) (*P* < 0.0001). The mean height growth was 1.9 cm/year for PFMI grade 5, and 0.3 cm/year for grade 6, with significant differences between these groups (*P* < 0.001).

Conclusions

PFMI allowed further categorization within Risser stage 4: PFMI grade 5 indicated remaining growth potential and risk of postbracing curve progression, whereas grade 6 indicated growth cessation. The combined use of Risser staging and PFMI, both evaluable through the same whole-spine radiograph, may provide a more accurate prediction of growth cessation.

## Introduction

Conservative and surgical treatments are the main categories for managing adolescent idiopathic scoliosis (AIS). Conservative treatments include bracing, exercise therapy, electrical stimulation, and traction therapy, with bracing being the most standard and evidence-based option [1-4].

Curve progression in AIS is predominantly correlated with maturation; the period of most substantial curve progression coincides with peak height velocity (PHV) [5,6] and gradually ceases upon the completion of skeletal maturation [7,8]. Consequently, the Scoliosis Research Society (SRS) developed guidelines for bracing indications based on growth potential and curve size [9]. However, the optimal time to terminate bracing is not well defined [4], and generally, weaning of bracing begins upon the completion of skeletal maturation [10]. According to the SRS, indications for skeletal maturation include a height growth of less than 1 cm at consecutive visits at intervals of six months or more, or if the height cannot be measured, Risser stage 4 or higher, and at least two years post-menarche in females [11,12]. Because height growth can only be measured retrospectively, skeletal maturity should be assessed using radiographic indicators. Risser staging [13] is the most commonly used assessment method [14-17]. However, Risser staging has several limitations including prolonged duration of Risser stage 4 [18], and failure in some adult patients to reach Risser stage 5 [4]. Therefore, various radiographic indicators based on different anatomical sites have been reported. These include the modified Sauvegrain method for the elbow joint and olecranon [19], Sanders staging for the hand [20], the DRU classification for the distal radius and ulna [21], and the thumb: ossification composite index for the thumb [22]. However, all of these methods require additional radiographs of the specific site to assess skeletal maturity.

The proximal femur maturity index (PFMI) reported by Cheung et al. in 2022 uses the femoral head, greater trochanter, and triradiate cartilage to classify skeletal maturity into seven grades (0-6), with higher grades indicating greater maturity. This method can be assessed using whole-spine radiographs, allowing monitoring of curve progression while avoiding additional radiation exposure for skeletal maturity assessment [23]. PFMI provides finely subdivided intervals between grades even in the later phases of skeletal maturation, making it a useful indicator of growth cessation. To our knowledge, no studies have reported on the post-bracing curve progression using PFMI. Therefore, this study aimed to clarify the utility of simultaneously using Risser staging and PFMI to evaluate the appropriate timing of growth cessation for terminating bracing, with the goal of preventing curve progression after bracing. To achieve this, we investigated the relationship between skeletal maturity at the end of bracing, as measured by Risser staging and PFMI, and post-bracing curve progression as well as height growth. 

## Materials and methods

Patients and methods

To clarify the relationship between post-bracing outcomes and skeletal maturity at the time of brace discontinuation in patients successfully treated with bracing, we first compared the average chronological age for each stage of the Risser staging and PFMI and assessed the correlation between these two classifications. Next, we examined the Risser staging and PFMI at the end of bracing and investigated their relationships with post-bracing curve progression. Finally, we analyzed the height growth rate at each stage to evaluate the timing of growth cessation in both classifications. 

**Figure 1:**
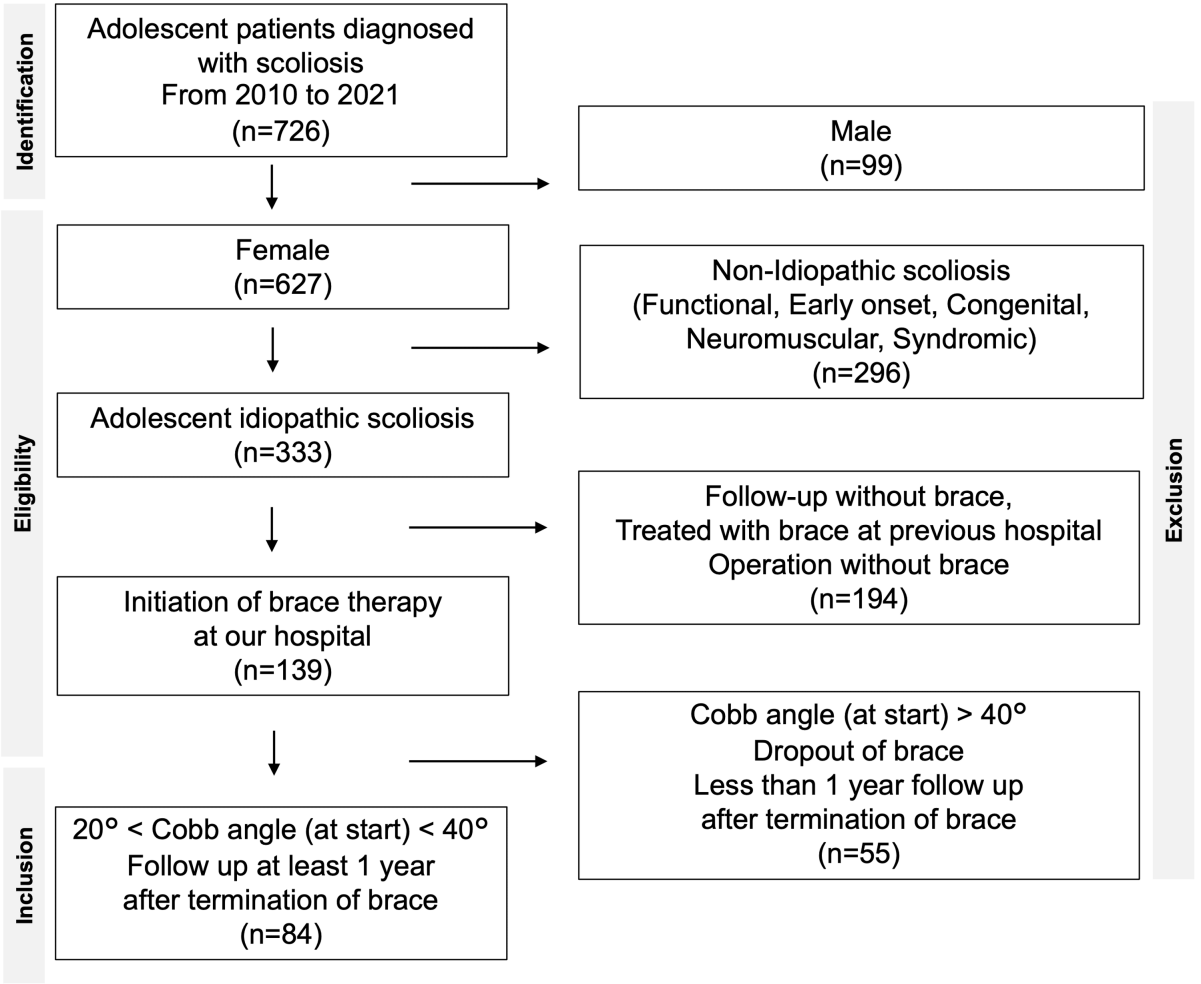
Flow chart of enrollment

Between April 2010 and March 2021, 84 of 726 patients diagnosed with scoliosis at Okayama University Hospital who met the following criteria were retrospectively reviewed. The inclusion criteria were as follows: (i) age 10-20 years at presentation, (ii) female, (iii) diagnosis of AIS, (iv) no history of previous scoliosis treatment at other hospitals and starting bracing at our hospital, (v) Cobb angle of 20°-40° at the start of bracing, and (ⅵ) follow-up of at least one year after bracing without surgical treatment (Figure 1). 

All patients were treated using the Osaka Medical College brace. The brace was applied when the main curve exceeded 20 degrees. The timing of brace termination was determined based on a comprehensive assessment of height growth, curve progression, skeletal maturity, and patient compliance.

Height and age at menarche were obtained from medical records. The height growth rate (cm/month) was calculated by dividing the height increase at each visit by the interval between visits. Radiographic parameters, including the Risser stage, PFMI, and Cobb angle of the main curve (thoracic spine, n = 54; thoracic/thoracolumbar spine, n = 30), were obtained from whole-spine standing radiographs taken at each visit. Post-bracing curve progression was measured by comparing the Cobb angle at the first visit after brace termination with that at the final follow-up or immediately before surgery in patients who received surgical treatment more than one year after discontinuing brace wear.

At the start of bracing, the mean age was 13.3 ± 1.1 years, and the mean Cobb angle was 31° ± 5°. At the end of bracing, the mean age was 15.3 ± 1.0 years, and the mean Cobb angle was 32° ± 6°. The curve progression during bracing was an average of 1° ± 5°, indicating that only patients for whom brace treatment was effective were included in the study. At the final follow-up, the mean age was 17.8 ± 1.6 years, and the mean Cobb angle was 34° ± 7°. The mean age at menarche was 12.5 ± 1.1 years. The mean duration of bracing was 2.0 ± 0.9 years, the mean duration from menarche to the end of bracing was 2.9 ± 0.9 years, and the mean follow-up duration after the end of bracing was 2.5 ± 1.1 years (Table 1).

**Table 1:** Baseline characteristics of included cases

	At the start of brace	At the end of brace	Menarche	At the last visit
Age (y)	13.3±1.1	15.3±1.0	12.5±1.1	17.8±1.6
Cobb (°)	31±5	32±6		34±7

This study was approved by the ethics committee of Okayama University (permission No. 2212-021).

Statistical analyses

Data were analyzed using GraphPad Prism version 10.2.0 (GraphPad Software, San Diego, CA, USA). Variables are presented as mean and standard deviation. Between-group comparisons were performed using the unpaired t-test, whereas comparisons among three groups were performed using one-way analysis of variance. Fisher’s exact test was used for the independent tests, and the Spearman correlation test was used for the correlation analysis. Statistical significance was set at P < 0.05.

## Results

Relationship among the Risser stage, PFMI, and chronological age

**Figure 2:**
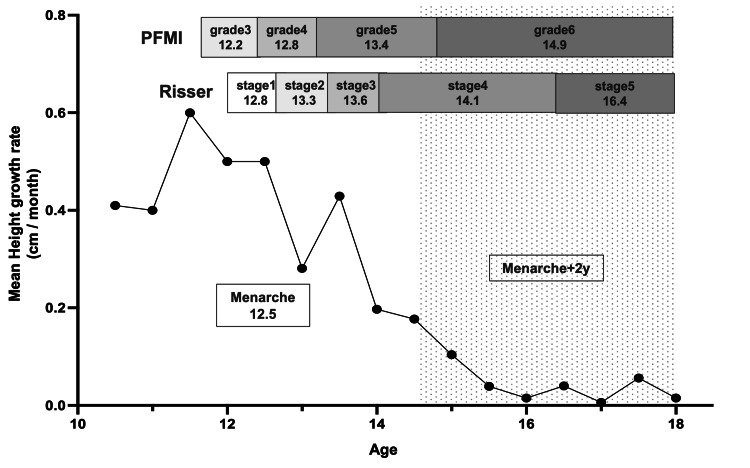
Relationship among Risser staging, proximal femur maturity index (PFMI) and chronological age The X-axis represents chronological age, and the Y-axis represents height growth. The number under each stage represents the average chronological age.

A positive correlation was observed between the Risser stage and PFMI (ρ = 0.7995). On average, it took 2.3 years to progress from Risser stage 4 (mean age, 14.1 ± 1.0 years) to stage 5 (mean age, 16.4 ± 1.1 years), which was longer than the transitions between other stages. Additionally, an average of 1.5 years was required to progress from PFMI grade 5 (mean age, 13.4 ± 0.9 years) to grade 6 (mean age, 14.9 ± 1.2 years). During the Risser stage 4 period, all cases reached PFMI grade 6, which is considered the completion of skeletal growth (Figure 2).

The mean age at menarche was 12.5 ± 1.1 years. All patients with Risser stage 5 and PFMI grade 6 had passed at least two years since menarche. Although more than two years had passed since menarche, some cases were still at Risser stage 4 and/or PFMI grade 5.

Relationship between skeletal maturation at bracing termination and post-bracing curve progression 

At the time of bracing termination, 85.7% (n = 72) of the patients were at Risser stage 4, whereas 14.3% (n = 12) were at stage 5. Overall, the curve progressed by an average of 1.5° ± 3.0° from the end of bracing to the final follow-up.

**Figure 3:**
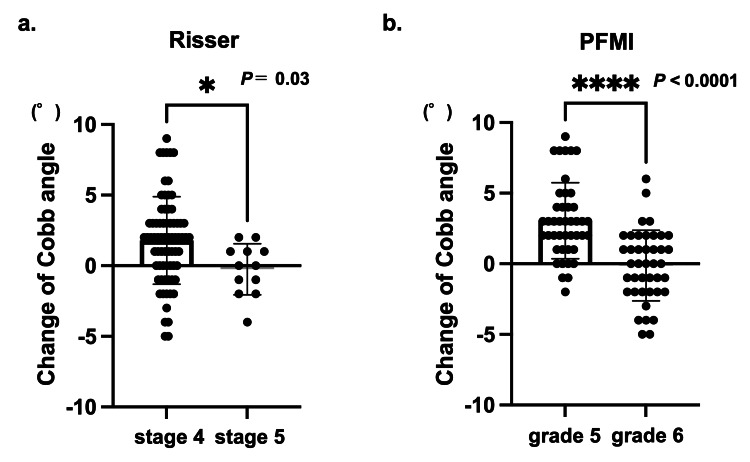
Postbracing curve progression a: Risser staging, b: proximal femur maturity index (PFMI) When brace treatment was terminated at Risser stage 4 or PFMI grade 5, the curve was significantly progressed compared to Risser stage 5 and PFMI grade 6, respectively. Statistical analysis was performed by unpaired t-test.

Among cases that terminated at Risser stage 4, the curve progressed an average of 1.8° ± 3.1°, whereas for those at stage 5, it was −0.3° ± 1.7°, indicating significant curve progression in cases that terminated at stage 4 (P = 0.03) (Figure 3A). Furthermore, 9.5% (n = 8) of the patients showed progression of ≥6°, and all patients completed bracing at stage 4.

At the end of bracing, PFMI grade 5 was observed in 51.2% (n = 43) of the patients and grade 6 in 48.8% (n = 41). The mean curve progression at the final follow-up was 3.0° ± 2.7° for grade 5 and 0.1° ± 2.3° for grade 6, indicating significant curve progression in grade 5 cases (P < 0.0001) (Figure 3B). Among the eight patients with progression of ≥6°, seven were classified as grade 5.

**Figure 4:**
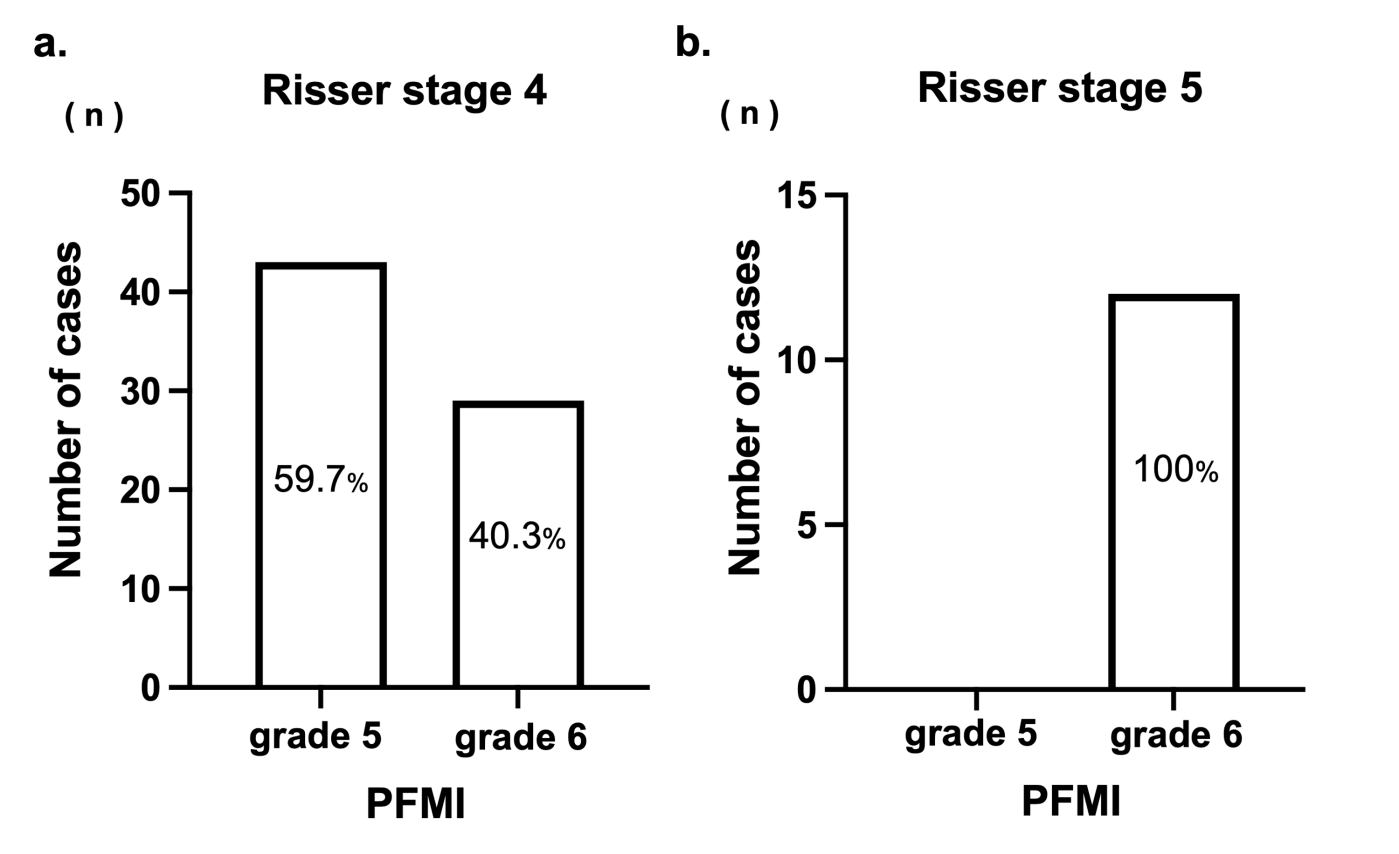
The ratio of proximal femur maturity index (PFMI) in Risser stage 4 and 5 at the end of brace a: Risser stage 4, b: Risser stage 5

When comparing the Risser stage and PFMI at the end of bracing, all cases in which bracing was terminated at Risser stage 5 coincided with PFMI grade 6. Among patients who completed bracing at Risser stage 4, 59.7% (n = 43) were classified as PFMI grade 5 and 40.3% (n = 29) were at grade 6 (Figure 4).

In patients with Risser stage 4, no significant difference in chronological age at brace termination, Cobb angle, follow-up duration, or duration from menarche to brace termination was found between PFMI grades 5 and 6. However, post-bracing curve progression was significantly worse in grade 5 (3.0° ± 2.7°) than in grade 6 (−0.1° ± 2.7°) (P < 0.0001; Table 2). 

**Table 2:** Characteristics of patients who completed bracing at Risser stage 4 Note: Statistical analysis was performed by unpaired t-test. * indicates significant difference (P < 0.05).

	n	Age (y) (at the end of brace)	Cobb (°) (at the end of brace)	Follow-up Duration (y)	Duration from menarche to brace off (y)	Curve progression(°) (after brace off)
Total	72	15.2±1.0	32.1±6.4	2.4±1.1	2.7±0.6	1.8±3.1
Grade 5	43 (59.7%)	15.1±1.0	32.5±6.7	2.6±1.1	2.8±0.6	3.0±2.7
Grade 6	29 (40.3%)	15.3±1.1	31.6±6.4	2.2±0.9	2.6±0.6	-0.1±2.7
p-value		ns	ns	ns	ns	* P < 0.0001

Among the cases that terminated bracing at Risser stage 4 and PFMI grade 5, 16.2% progressed to >6°. By contrast, only 3.4% of patients inRisser stage 4 and PFMI grade 6 experienced progression of >6° (P = 0.132).

Relationship between height growth, Risser stage, and PFMI

**Figure 5:**
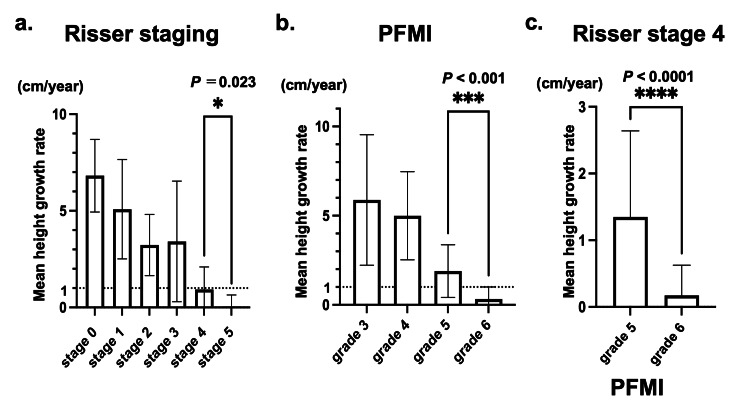
Average annual height growth a, b: There was a significant difference between Risser stage 4 and 5, as well as proximal femur maturity index (PFMI) grade 5 and 6. Statistical analysis was performed by one-way ANOVA. c: Patients who terminated bracing at Risser stage 4, the mean height growth after brace termination was PFMI grade 6: 0.2±0.4 cm, grade 5: 1.3±1.3 cm with significance (unpaired t-test).

The annual height growth rates at Risser stages 4, 5, PFMI grade 5, and grade 6 were 0.9 ± 1.2, 0.1 ± 0.6, 1.9 ± 1.5, and 0.3 ± 0.7 cm/year, respectively. Both methods showed a stepwise decrease in height growth with higher stages. Additionally, a significant difference in height growth rate was found between Risser stages 4 and 5 and between PFMI grades 5 and 6 (P = 0.023, P < 0.001, respectively) (Figure 5A-5B). Interestingly, the average height growth at Risser stage 4 was <1 cm/year, indicating a general cessation of growth. However, further analysis based on PFMI of patients who discontinued bracing at Risser stage 4 revealed additional insights. For these patients, the mean annual height growth at PFMI grade 6 was 0.2 ± 0.4 cm, indicating the end of the growth period, whereas at PFMI grade 5 it was 1.3 ± 1.3 cm, suggesting that patients at Risser stage 4 and PFMI grade 5 still had growth potential (P < 0.0001) (Figure 5C).

## Discussion

The effectiveness of bracing for AIS has been recognized by the widely cited Bracing in Adolescent Idiopathic Scoliosis Trial study (BrAIST) [2] and more recently in a review that included only randomized controlled trials by Zhang et al. [3]. However, bracing is always associated with radiation exposure [17,24,25]. Other concerns such as skin irritation and psychological distress also exist. Therefore, predicting the period during which the curve will likely progress and limiting bracing to that period to reduce unnecessary brace treatment is necessary [4]. Given the unclear timing of complete skeletal maturity and the absence of well-defined brace weaning criteria, determining the appropriate timing of brace termination is challenging [4]. This study aimed to clarify whether PFMI, which can be evaluated using a single whole-spine radiograph, can complement Risser staging by providing a more detailed assessment of the later phases of skeletal maturity.

Cheung et al. reported that PFMI correlated well with Risser staging (τb = 0.743) [22], and a similar correlation was observed in this study (ρ = 0.7995). Regarding the relationship with chronological age, Hoppenfeld et al. reported that the duration of Risser stage 4 was relatively long (mean, 2.5 years) [25]. This study confirmed the long duration of stage 4, with an average of 2.3 years, and included PFMI grades 5 and 6 during this period. During Risser stage 4, maturity progressed from PFMI grade 5 to grade 6, with an average of 1.5 years to reach Risser stage 5.

The curve progression during the bracing period for the cases included in this study averaged 1°. Cases that underwent surgery either during the bracing treatment or within one year after its termination were excluded, enabling an examination of post-curve progression in patients who were considered to have been successfully treated with bracing. Little curve progression was observed when bracing was terminated at the final phases of Risser staging and PFMI. By contrast, significant progression was observed in patients who terminated at Risser stage 4 and PFMI grade 5. Furthermore, among the cases terminated at Risser stage 4, those at PFMI grade 5 showed significantly more curve progression than those at grade 6. This indicates that within the relatively long period of Risser stage 4, patients with PFMI grade 5 are at risk of curve progression and should be carefully monitored after bracing. Cheung et al. reported that Risser staging alone is insufficient for brace weaning and suggested that Sanders stage 8 and radius grade 10/ulna grade 9 are the most appropriate weaning time points [4]. However, the Sanders and DRU classifications require additional radiographs to assess skeletal maturity. Therefore, PFMI, which can be assessed using whole-spine radiography, is an excellent method for reducing radiation exposure.

In this study, the average height growth rate at Risser stage 4 was <1 cm/year, consistent with the general standard that Risser stage 4 indicates complete skeletal maturity. However, when further classified using PFMI, PFMI grade 5 within Risser stage 4 showed growth potential. This finding is supported by previous reports indicating that histological analysis of vertebral endplates from patients with AIS showed growth activity in 10 out of 14 Risser stage 4 cases [26], and another study reported that 75.2% of children at Risser stage 4 exhibited an average growth of 1.75 cm (range, 0.5-8.9 cm) [18].

In summary, these results indicate that even within Risser stage 4, PFMI grade 5 cases have growth potential and risk of curve progression. This study suggests that confirming Risser stage 4 and PFMI grade 6 could be reliable indicators of growth cessation, thereby ensuring a safer weaning process. By combining the Risser stage with PFMI, the final phase of skeletal maturity can be predicted accurately without additional radiation exposure.

This study has several limitations. First, selection bias is possible owing to the small sample size and retrospective design. Although the incidence of scoliosis is roughly the same in both sexes, the risk of curve progression is 10 times higher in females [27]. Additionally, the growth speed and timing of skeletal maturation vary between sexes; therefore, males were not included in this study. Second, the evaluation of radiographic parameters was conducted solely by the author; thus, interobserver reliability could not be assessed. However, Wang et al. demonstrated that the proposed PFMI has good inter- and intraobserver reliability [28]. Finally, the decision on the timing of brace weaning was not based on uniform criteria, as each attending physician made a comprehensive judgment based on height growth, curve progression, skeletal maturity, and patient compliance.

## Conclusions

This study investigated the relationship among height growth, Risser stage, and PFMI in patients with AIS. PFMI allowed us to categorize the period of Risser stage 4 into PFMI grade 5, which still had growth potential and a risk of postbracing curve progression, and grade 6, which indicated growth cessation. The combined use of Risser staging and PFMI on the same whole-spine radiograph provides an additional measure of growth cessation without requiring additional radiation exposure. In particular, the assessment of PFMI is valuable at Risser stage 4, which represents the later stage of skeletal maturity.
